# Rational Approach for Evaluating Fire Resistance of Prestressed Concrete Beams Strengthened with Fiber-Reinforced Polymers

**DOI:** 10.3390/polym17202773

**Published:** 2025-10-16

**Authors:** Venkatesh Kodur, Tejeswar Rayala, Hee Sun Kim

**Affiliations:** 1 Department of Civil and Environmental Engineering, Michigan State University, East Lansing, MI 48824, USA; rayalate@msu.edu; 2 Architectural and Urban Systems Engineering, Ewha Womans University, Seoul 03760, Republic of Korea; hskim3@ewha.ac.kr

**Keywords:** prestressed concrete beams, fiber-reinforced polymers, fire resistance, FRP strengthening, rational fire design approach

## Abstract

A rational approach is proposed for evaluating the fire resistance of fiber-reinforced polymers (FRP)-strengthened prestressed concrete (PC) beams. This approach expands on conventional fire design principles for PC beams, while incorporating the effects of FRP reinforcement and fire insulation into strength calculations under fire exposure. Simplified equations are utilized to evaluate the cross-sectional temperature distribution in fire-exposed FRP-strengthened PC beams, considering both insulated and uninsulated scenarios. These cross-sectional temperature profiles are then utilized to evaluate the reductions in the strengths of concrete, steel, and FRP based on their temperature-dependent mechanical properties. The moment capacity of the FRP-strengthened PC beams is determined at various fire exposure durations by applying force equilibrium and strain compatibility principles, assuming a full bond with no relative slip between the FRP and the concrete interface under fire exposure. The critical strength limit state is applied at each time interval to determine the failure state of the FRP-strengthened PC beam, with the final time to failure considered to be the fire resistance of the beam. The proposed approach is validated by comparing its results with available test data from FRP-strengthened reinforced concrete (RC) beams. The validated model is applied to evaluate critical parameters governing the fire resistance of FRP-strengthened PC beam. The results show that, without fire insulation, FRP-strengthened PC beams undergo a significant reduction in moment capacity early into fire exposure and fail within 75 min due to the rapid strength degradation of both the CFRP and the prestressing steel. In contrast, the application of 25 mm thick fire insulation allows these beams to retain a substantial portion of their load-bearing capacity for up to 3 h of fire exposure.

## 1. Introduction

Prestressed concrete (PC) structures are widely used in modern construction due to their durability and ability to span longer distances with reduced cross-sectional dimensions. However, over time, these structures experience strength degradation caused by factors such as loss of prestress, poor maintenance, corrosion of prestressing steel strands, concrete aging, and exposure to aggressive environmental conditions. Additionally, increased loading demands, changes in building use, changes in occupancy type, design deficiencies, and updated building codes necessitate retrofitting and strengthening of the structure to ensure continued safety and performance. Traditional strengthening methods for PC structures include enlarging concrete sections, applying steel jackets, bonding steel plates, and using external post-tensioning with steel tendons [1]. Although effective, these techniques add significant weight, alter structural geometry, and are prone to corrosion, resulting in higher maintenance costs and complex installation processes. In contrast, fiber-reinforced polymers (FRPs) have emerged as a popular alternative for strengthening PC structures due to their high strength-to-weight ratio, excellent corrosion resistance, ease of application, and minimal impact on the structure’s appearance and weight.

Extensive research has been conducted on the mechanical performance of FRP-strengthened PC structures at ambient temperature, with various studies demonstrating significant improvements in structural capacity. The strengthening of prestressed bridge slabs was experimentally investigated using five FRP systems, including near-surface mounted (NSM) bars and externally bonded (EB) carbon fiber-reinforced polymers (CFRP) [2]. The study found that CFRP reinforcement strengthening enhanced stiffness and increased ultimate load capacity of slabs by up to 50%. Similarly, an experimental investigation on the static loading behavior of prestressed concrete bridge girders revealed that CFRP strengthening improved flexural capacity by up to 73% while maintaining ductility [3,4]. Additionally, the findings indicated that CFRP significantly reduced crack spacing and widths, with crack widths decreasing by up to 10% compared to un-strengthened girders. Subsequently, five full-scale PC girders with varying levels of tendon damage and CFRP strengthening were experimentally tested [5]. The study found that 17% and 33% strand loss led to 20% and 26% reductions in flexural capacity, respectively. CFRP laminates with U-wrap anchorage restored up to 16% of capacity but resulted in brittle failure. Furthermore, an experimental study on the strengthening of 60-year-old PC bridge deck units using adhesively bonded CFRP plates demonstrated a 10% increase in load-carrying capacity at the serviceability limit and a 54% improvement in ultimate strength [6].

Despite the numerous advantages of FRP composites, they exhibit significant performance limitations when exposed to elevated temperatures, making fire resistance a major concern in structural applications [7,8,9]. The primary limitation arises from the polymer matrix, which is highly susceptible to thermal degradation. As temperatures approach the glass transition temperature ($T_{g}$), typically from 65 °C to 100 °C, the polymer matrix softens, reducing its ability to transfer shear stress between fibers and leading to a decline in FRP mechanical properties such as tensile strength and stiffness [10,11]. Further exposure to temperatures from 300 °C to 500 °C causes the decomposition of the resin, releasing heat, smoke, soot, and harmful volatiles, which not only exacerbates the degradation of mechanical properties but also poses environmental and safety concerns [12]. At these temperatures, chemical bonds within the polymer structure break down, leading to fiber–matrix debonding and structural failure [13]. Consequently, the lack of inherent fire resistance in FRP materials necessitates additional protective measures when used in load-bearing structural applications, as their failure can occur early into fire exposure if not adequately insulated, specifically in externally bonded FRP systems [14].

To address the fire-induced vulnerabilities of FRP-strengthened structural members, extensive research has been conducted to evaluate their behavior under elevated temperature exposure and to develop strategies to enhance their fire resistance. Experimental studies demonstrated that CFRP-strengthened rectangular RC beams with 25 mm spray-applied Tyfo^®^ WR can withstand failure under design fire conditions, including three hours of ASTM E119 growth phase followed by a decay (cooling) phase [15]. Additionally, experimental and numerical investigations on CFRP-strengthened RC beams evaluated the effectiveness of calcium silicate(CS) boards and vermiculite/perlite (VP) mortar as fire protection [16,17]. The findings revealed that unprotected CFRP debonded from RC beams after 23 min, whereas 25 mm and 40 mm insulation extended failure times to 60–89 min and 137–167 min, respectively, with VP mortar proving more effective than CS boards in reducing temperatures at the CFRP–concrete interface. Likewise, experimental and numerical studies demonstrated that CFRP-strengthened RC T-beams with 19 mm and 32 mm spray-applied fireproofing on the bottom and sides sustained service load levels for up to four hours under ASTM E119 fire exposure [18].

Despite these advancements, experimental and numerical studies on the fire resistance of FRP-strengthened beams remain complex, time-consuming, and require advanced modeling techniques. To address this limitation, a rational design method was proposed that incorporates the contribution of FRP, the effects of fire insulation, and temperature-dependent material degradation into fire resistance calculations [19]. Furthermore, the accuracy of this approach has been validated through comparisons with fire test data and finite element analysis (FEA) results, demonstrating a margin of error within 10%, confirming its applicability and reliability for structural fire design. Following this development, a detailed review was presented on the fire performance of FRP-incorporated concrete structures, with particular focus on the thermal, mechanical, bond, and combustibility properties of FRP materials at both room and elevated temperatures [10]. The review incorporates findings from experimental studies, numerical simulations, and existing code provisions to provide a comprehensive overview of the current understanding of fire behavior in FRP-strengthened members. Based on these studies, it is evident that while fire resistance ratings of up to four hours can be achieved with appropriate fire protection, current design practices do not adequately account for the degradation of FRP properties at elevated temperatures. The findings also highlight the importance of developing rational fire design approaches that accurately reflect the behavior of FRP systems under fire exposure.

The fire resistance of FRP-strengthened RC beams has been extensively studied over the past few decades, with numerous studies focusing on their structural behavior under elevated temperatures. However, there is a noticeable lack of research specifically addressing FRP-strengthened PC beams, as most existing studies have focused primarily on their performance under ambient conditions without considering the impact of fire. There is a significant difference in the degradation of mechanical properties of prestressing steel compared to conventional reinforcing steel bars under elevated temperature exposure. Prestressing steel, due to its composition, tends to lose strength more quickly, which can cause earlier and more severe failure. Experimental studies have reported that the ultimate and yield strength of low-relaxation seven-wire prestressing strands degrade more rapidly than reinforcing steel bars over the 20–800 °C temperature range [20]. Additionally, a strength reduction of approximately 50% was observed around 450 °C. Since both FRP materials and prestressing tendons experience substantial degradation at elevated temperatures, further investigation is essential to understand their combined effect on structural behavior, failure mechanisms, and potential fire protection strategies.

This paper addresses the existing knowledge gap by extending the rational approach, previously developed for FRP-strengthened RC beams, to evaluate the fire resistance of CFRP-strengthened PC beams. The methodology is first validated against available experimental and numerical data on FRP-strengthened RC beams and subsequently applied to PC beams to quantify their fire performance. A comprehensive parametric study is conducted to evaluate the effects of insulation thickness, concrete cover depth, and strengthening configuration on the fire resistance of CFRP-strengthened PC beams, and the resulting findings, methodological limitations, and design implications are presented to guide their safe application in fire-prone environment.

## 2. Rational Approach for Evaluating Structural Behavior Under Fire Conditions

A systematic approach for evaluating the fire resistance of FRP-strengthened PC beams is developed in this paper, based on established fire design principles for conventional PC beams, incorporating the influence of FRP reinforcement and fire insulation in fire resistance evaluation. This approach consists of two fundamental steps: (1) analyzing the temperature distribution resulting from fire exposure within the concrete, FRP, and prestressing tendons of the strengthened PC beam, and (2) computing the reduced moment capacity at the critical cross-section for varying durations of fire exposure, with due consideration to temperature induced degradation in strength properties of all constituent materials.

**Figure 1:**
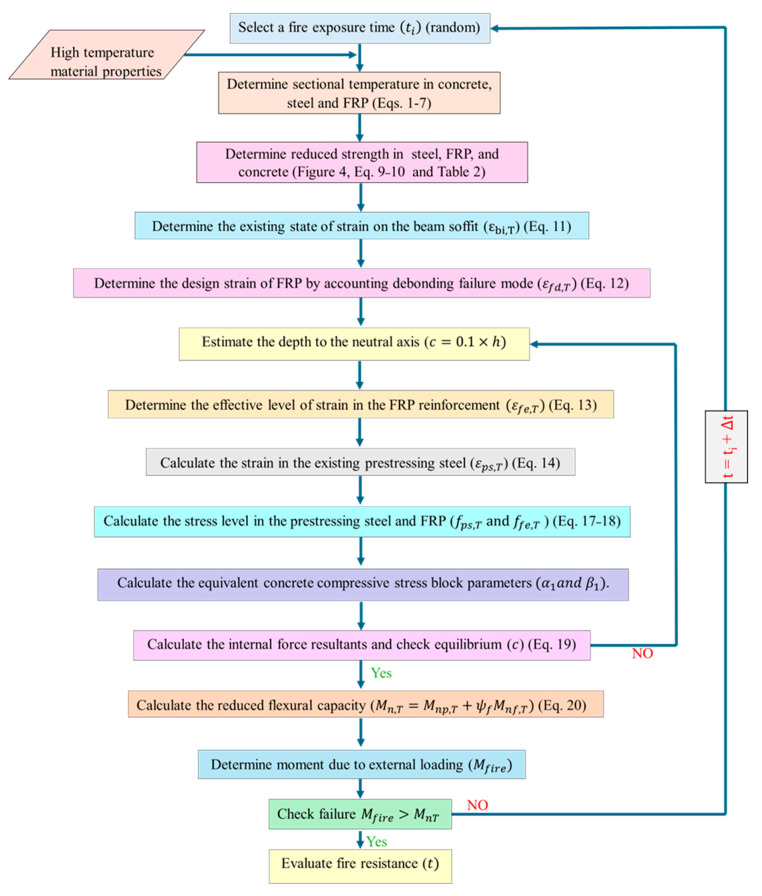
A flowchart showing steps in the rational methodology for assessing fire resistance of FRP-strengthened PC beam.

The flowchart in Figure 1 illustrates the step-by-step procedure for evaluating the fire resistance of an FRP-strengthened PC beam. The evaluation process involves iterative calculations conducted at various time increments until failure occurs under fire exposure. Failure is defined as the time step at which the moment capacity at the critical section falls below the applied moment due to loading. The total time elapsed until this failure step is recorded as the fire resistance of the beam. Smaller time increments, such as one minute, are recommended to improve the accuracy of failure prediction, since CFRP and prestressing steel undergo rapid strength and stiffness degradation once critical temperatures are reached.

The first step in the analysis involves determining temperature distributions within the FRP, prestressing steel, and concrete for a specified fire exposure duration using simplified equations, accounting for both insulated and uninsulated FRP-strengthened PC beams. Based on the sectional temperatures, the resulting degradation in material properties, including reductions in strength and stiffness, is determined. The strain in both the FRP and prestressing steel is then assessed while considering the effects of potential debonding failure and rupture. Corresponding stresses are computed using the reduced strength and modulus properties of the FRP and prestressing steel.

The concrete compressive stress block parameters are subsequently calculated to establish equilibrium within the section. Once the force equilibrium is attained, the reduced flexural capacity of the FRP-strengthened PC beam is determined by incorporating the strength contributions of the prestressing tendons, FRP reinforcement and concrete. The final step involves comparing the reduced moment capacity with the applied moment demand under fire conditions, with the final time at which failure occurs being deemed as the fire resistance of the beam.

## 3. Analysis of Cross-Sectional Temperatures

The fire resistance of FRP-strengthened PC beam is significantly affected by the degradation of strength in the FRP reinforcement, prestressing steel, and concrete due to elevated temperatures. Therefore, accurate prediction of cross-sectional temperature distribution at various fire exposure durations is essential for evaluating the structural response of the beam under fire conditions. For an uninsulated FRP-strengthened PC beam, the temperature distribution across the cross-section remains nearly the same as that of an un-strengthened PC beam. This is because externally bonded FRP reinforcement has a relatively much smaller cross-sectional area compared to concrete, resulting in a minor impact on heat transfer within the beam. A set of simplified equations was developed to estimate cross-sectional temperatures in RC members exposed to standard fire conditions [21]. These equations have been validated through FEA results and fire test data, demonstrating accuracy within a ±10% margin, particularly for temperatures in the range of 300–700 °C. Since PC and RC beams exhibit similar thermal responses, the same temperature prediction equations are applied in this study for FRP-strengthened PC beams.

**Figure 2:**
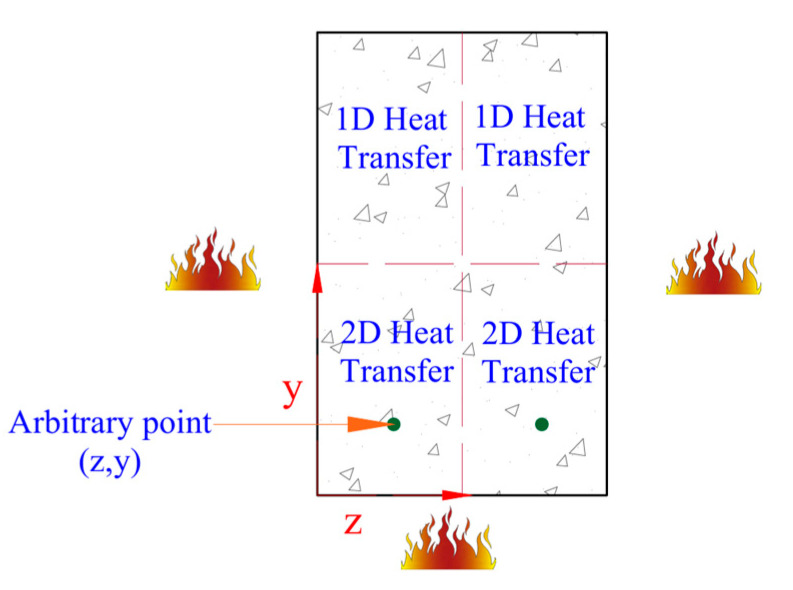
Fire-induced heat transfer mechanisms in a 3-sided exposed concrete beam.

The temperature ($T_{c})\;$at any point within the cross-section of a concrete beam, represented by coordinates y and z in Figure 2, can be determined for a specified fire exposure duration using this method and the below equations.

For one-dimensional heat transfer, we use

(1) $$T_{c}=c_{1}\cdot \eta_{z}\cdot \left(at^{n}\right)$$

For two-dimensional (2D) heat transfer, we use

(2) $$T_{c}=c_{2}\left[\left(-1.481\cdot \left(\eta_{z}\cdot \eta_{y}\right)+0.985\cdot \left(\eta_{z}+\eta_{y}\right)+0.017\right)\right]\left(at^{n}\right)$$

where

(3) $$\eta_{z}=0.155\;In\frac{t}{z^{1.5}}-0.348\sqrt{z}-0.371$$

(4) $$\eta_{y}=0.155\;In\frac{t}{y^{1.5}}-0.348\sqrt{y}-0.371$$

The parameter t represents duration of fire exposure in hours, z and y indicate the distance from a given point within the concrete section to the fire-exposed surface (m), and $at^{n}$ represents the fire temperature according to ISO 834 or ASTM E119 standard fire conditions. The coefficients a, n, $c_{1}$, and $c_{2}$ used in the temperature prediction equations are taken from Table 1. Equations (1)–(4) are applicable for estimating cross-sectional temperatures in FRP-strengthened PC beams without fire insulation.

**Figure 3:**
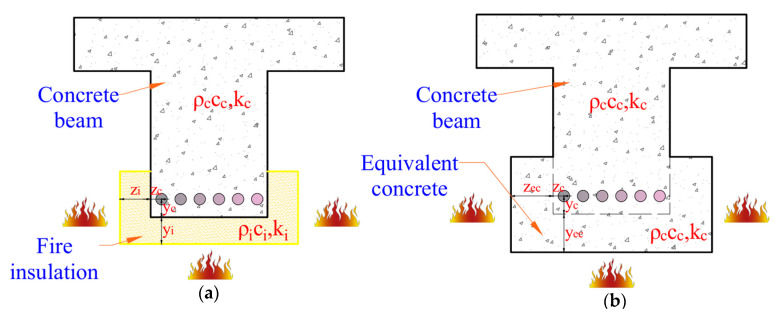
Equivalent concrete depth approach for predicting cross-sectional temperatures. (**a**) insulated concrete section and (**b**) equivalent concrete section.

For an FRP-strengthened PC beams with fire insulation, the temperatures in concrete, prestressing steel, and FRP reinforcement are significantly reduced due to the protective insulating effect of the fire insulation layer. In these insulated beams, thermal behavior is primarily influenced by the properties of both insulation and concrete. The temperature distribution within an insulated PC section can be determined using the same equations [Equations (1) and (2)] applied to uninsulated PC beams, by converting the insulation layer into an equivalent concrete layer to account for its thermal resistance. Since fire insulation materials commonly used in protection possess much lower thermal conductivity and higher specific heat capacity than concrete, the insulated region experiences a significant reduction in heat transfer. To account for this in thermal analysis, the insulation layer is transformed into an equivalent concrete layer that provides the same thermal resistance. Due to the effective insulating properties, the resulting equivalent concrete section becomes significantly enlarged to match the resistance, as illustrated in Figure 3b.

A U-shaped fire layer is applied to a PC beam, as shown in Figure 3a, with insulation thicknesses $z_{i}$ on the sides and $y_{i}$ on the bottom. When the insulation layer is substituted with an equivalent concrete layer of thickness $z_{ec}$ on the sides and $y_{ec}$ on the bottom, as illustrated in Figure 3b, the temperature distribution within the beam’s cross-section should remain unchanged. By applying fundamental heat transfer principles and one-dimensional heat transfer equation [Equation (1)], the relationship between the fire insulation thickness and the corresponding equivalent concrete thickness can be derived [21]:

(5) $$\frac{z_{ec}}{z_{i}}=\sqrt[{\alpha }]{t}\cdot \sqrt[{\beta }]{\left(\frac{k_{c}}{{\left(\rho c\right)}_{c}}\right)\left(\frac{{\left(\rho c\right)}_{i}}{k_{i}}\right)}$$

where α and β are regression coefficients, while $k_{c},{\left(\rho c\right)}_{c}$ and $k_{i},{\left(\rho c\right)}_{i}$ are thermal conductivity and heat capacity of concrete and insulation, respectively.

Through nonlinear regression analysis of a comprehensive dataset generated using FEA, the regression coefficients α and β were determined as 4.5 and 1.75, respectively [22]. The accuracy of these coefficients, obtained from regression analysis, falls within a 5–15% range, depending on the sectional dimensions of the beam and concrete properties. By applying the equivalent concrete depth method, the distance from the outer edge of the insulation to the fire-exposed surface ($z_{c}^{\prime }$ and $y_{c}^{\prime })$ can be determined using the following equations.

(6) $$z_{c}^{\prime }=z_{c}+z_{ec}=z_{c}+z_{i}\;\sqrt[{4.5}]{t}\cdot \sqrt[{1.75}]{\left(\frac{k_{c}}{{\left(\rho c\right)}_{c}}\right)\left(\frac{{\left(\rho c\right)}_{i}}{k_{i}}\right)}$$

(7) $$y_{c}^{\prime }=y_{c}+y_{ec}=y_{c}+y_{i}\;\sqrt[{4.5}]{t}\cdot \sqrt[{1.75}]{\left(\frac{k_{c}}{{\left(\rho c\right)}_{c}}\right)\left(\frac{{\left(\rho c\right)}_{i}}{k_{i}}\right)}$$

These equivalent concrete depths ($z_{c}^{\prime }$ and $y_{c}^{\prime }$) can be substituted into Equations (1)–(4) by replacing z and y to evaluate the cross-sectional temperature rise in an insulated PC member.

## 4. Degradation of Material Properties

**Figure 4:**
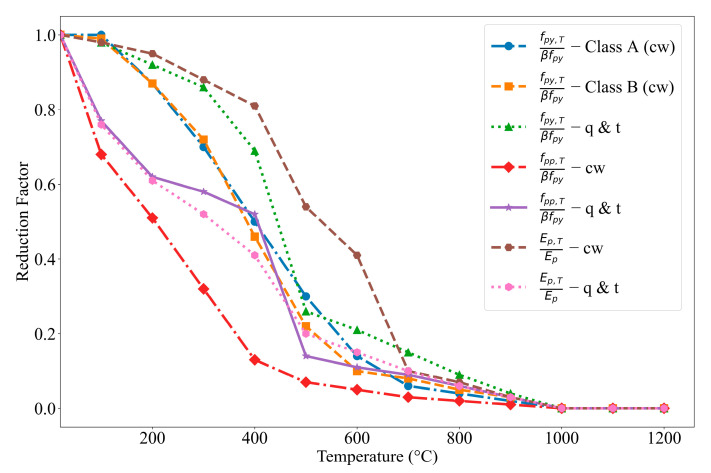
Stress–strain relationship parameters for prestressing steel at elevated temperatures adopted from Eurocode 2.

Knowing the cross-sectional temperatures within a PC beam, the impact of temperature on the strength degradation of concrete, prestressing steel, and FRP can be determined. Prestressing steel undergoes a more rapid reduction in strength and modules compared to conventional steel reinforcement, making it particularly vulnerable to fire exposure. Various models have been developed to characterize the temperature-dependent behavior of prestressing steel, with well-established relationships available for predicting strength and elastic modulus degradation. In this study, the temperature-strength and stiffness relationships specified in Eurocode 2 [23] (Figure 4) are adopted to evaluate the strength and modulus reductions in prestressing steel at different temperature levels.

In Figure 4, cw refers to cold-worked (wires and strands), q & t refers to quenched and tempered (bars), and β is a factor calculated based on Equation (8). The parameters $\epsilon_{ud},\epsilon_{uT},f_{p0,1y},\;f_{py}$ and $E_{p}\;$are material properties at room temperature as per Eurocode 2 [23].

(8) $$\beta =\left\{\begin{array}{c} \left(\frac{\frac{\epsilon_{ud}-f_{po,1y}}{E_{p}}}{\frac{\epsilon_{uT}-\epsilon_{po,1y}}{E_{p}}}\right)\times \left(\frac{f_{py}-f_{p0,1y}}{f_{py}}\right)+\frac{f_{p0,1y}}{f_{py}}\;\mathrm{Class}\;A\\ 0.9\;\mathrm{Class}\;B \end{array}\right\}$$

Extensive research on externally bonded FRP laminates has demonstrated that FRP reinforcement undergoes significantly higher strength and stiffness degradation than reinforcing steel upon reaching its glass transition temperature ($T_{g}$). Through experimental and modeling studies, researchers have developed empirical relationships to quantify the reduction in strength and modules for different FRP types at elevated temperatures. The strength and modulus reduction relationships [Equations (9) and (10)] for externally bonded CFRP laminates at elevated temperatures, developed in previous studies, are currently utilized in evaluating fire resistance [14]. Similar relationships for other types of FRP, such as AFRP and GFRP, can be found in related research [19].

Strength reduction factor:

(9) $$f_{f,T}=f_{f,20{}^{\circ}c}\left[\left(\frac{1-a_{\sigma }}{2}\right)\tanh\left(-b_{\sigma }\left(T-c_{\sigma }\right)\right)+\left(\frac{1+a_{\sigma }}{2}\right)\right]\;a_{\sigma }=0.1;b_{\sigma }=5.83\times 10^{-3};c_{\sigma }=339.54$$

Elastic modulus reduction factor:

(10) $$E_{f,T}=E_{f,20{}^{\circ}c}\left[\left(\frac{1-a_{E}}{2}\right)\tanh\left(-b_{E}\left(T-c_{E}\right)\right)+\left(\frac{1+a_{E}}{2}\right)\right]\;a_{E}=0.05;b_{E}=8.68\times 10^{-3};c_{E}=367.41$$

Concrete generally exhibits good performance at elevated temperatures due to its low thermal conductivity and high heat capacity, which slows down heat penetration and delay strength loss. Unlike steel reinforcement, concrete retains a significant portion of its mechanical properties under fire exposure, especially in its inner core, where temperatures rise more gradually. However, longer exposure to high temperatures leads to progressive strength degradation, particularly in the outer layers of concrete elements. To account for strength degradation due to temperature exposure, Eurocode 2 [23] introduces the effective concrete width approach. This method takes into account the contribution of concrete in the portion of the compression zone width where the temperature remains below 500 °C, while the strength contribution of concrete in the outer concrete layers exceeding 500 °C is neglected.

**Figure 5:**
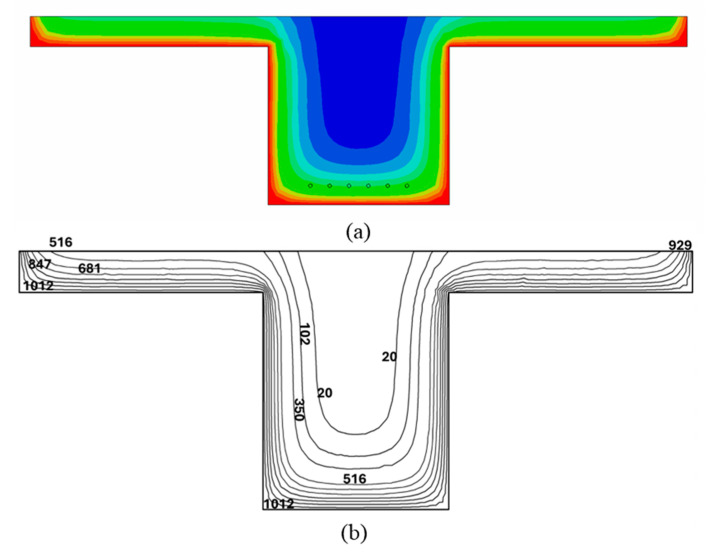
(**a**) Temperature distribution from ABAQUS and (**b**) temperature contours of PC T-Beam after 4 h of ASTM E119 fire exposure.

However, Eurocode 2 [23] does not specify values for effective width reduction in PC T-beams, resulting in a lack of predefined reduction factors for these elements. Although simplified cross-sectional temperature equations [Equations (1)–(4)] are available, they were not used in this study because they provide temperature values only at specific points within the section, making it difficult to construct a complete temperature gradient curve throughout the cross-section of the beam. To achieve better accuracy, a finite element analysis (FEA) using ABAQUS was conducted to evaluate the thermal response of the PC beam cross-section at specified fire exposure durations, following the simulation process demonstrated in previous research [24]. The temperature-dependent thermal properties of concrete, such as thermal conductivity and specific heat, were adopted from Eurocode 2 [23]. The temperature gradient curves shown in Figure 5b were analyzed across the cross-section, and the outer layer elements in the upper half of the beam within the compression zone, where temperatures exceeded 500 °C, were excluded from the total beam width. The effective reduction factors were then calculated and presented in Table 2.

## 5. Determining Moment Capacity at a Specified Fire Exposure Duration

Knowing the temperature-induced degradation of material properties, including the yield stress $\left(f_{y,T}\right)$, ultimate stress $\left(f_{u,T}\right)$ and Young’s modulus $\left(E_{p,T}\right)\;$of prestressing steel, along with the ultimate stress $\left(f_{f,T}\right)$ and Young’s modulus $\left(E_{p,T}\right)$ of FRP reinforcement and $f_{c}^{\prime }$ of concrete, the flexural capacity of FRP-strengthened PC beams at a specific fire exposure time can be determined by using a methodology similar to that used for room-temperature conditions, as outlined in ACI 440.2R-17 [8] and ACI [25]. According to ACI 440.2R-17, failure in an FRP-strengthened beam section at ambient temperature can occur through multiple mechanisms. These include concrete crushing in compression before the prestressing steel reaches its yield strength, yielding of the steel in tension followed by rupture of the FRP laminate, and yielding of the steel in tension leading to concrete crushing. Additionally, failure under fire exposure may result from shear or tensile delamination of the concrete cover, known as cover delamination, or debonding of the FRP reinforcement from the concrete substrate. These failure modes are incorporated in the analysis for calculating the flexural capacity of the beam.

Initially, the strain in the concrete substrate $\left(\varepsilon_{bi,T}\right)$ is determined and excluded from the effective strain in FRP reinforcement. This initial strain is obtained through an elastic analysis of the existing member, considering all loads present at the time of FRP installation. For this analysis, an uncracked section is assumed, considering only the dead load acting on the beam during FRP application, and the initial strain in the concrete substrate is calculated as:

(11) $$\varepsilon_{bi,T}=\frac{-P_{e,T}}{E_{c}A_{cg}}\left(1+\frac{ey_{b}}{r^{2}}\right)+\frac{M_{DL}y_{b}}{E_{c}I_{g}}$$

where $P_{e,T}$ = effective prestressing force which depends on strength degradation of prestressing steel; $E_{c}$ = modulus of elasticity of concrete; $A_{cg}$ = gross cross-sectional area; e = eccentricity of prestressing force; $y_{b}$ = distance from extreme bottom fiber to the section centroid; r = radius of gyration; $M_{DL}$ = moment due to dead load; and $I_{g}$ = gross moment of inertia.

At the section where the externally bonded FRP reinforcement end (anchorage points), failure due to FRP debonding may occur. To prevent intermediate crack-induced debonding, the effective strain in the FRP reinforcement should be restricted to the debonding strain $\left(\varepsilon_{fd,T}\right)$ threshold, which is determined using the following calculation [8]:

(12) $$\varepsilon_{fd,T}=0.41\sqrt{\frac{f_{c}^{\prime }}{nE_{f,T}t_{f}}}\leq 0.9\varepsilon_{fu}$$

where $f_{c}^{\prime }\;$= compressive strength of concrete (MPa); n = number of layers of FRP reinforcement; $t_{f}$ = thickness of FRP reinforcement; and $\varepsilon_{fu}$ = rupture strain of FRP reinforcement.

The effective strain in the FRP reinforcement can be estimated using an initial approximation of the neutral axis depth. The maximum strain that can develop in the FRP reinforcement is governed by one of three failure conditions: the strain at which concrete crushing occurs, the strain at which FRP rupture happens, or the strain at which FRP debonding from the substrate takes place. The effective strain $\left(\varepsilon_{fe,T}\right)$ in the FRP reinforcement at the ultimate limit state can be calculated as:

(13) $$\varepsilon_{fe,T}=\varepsilon_{cu}\left(\frac{h-c}{c}\right)-\varepsilon_{bi,T}\leq \varepsilon_{fd,T}$$

where h = effective depth of FRP reinforcement; c = neutral axis depth $\left(0.1\times h\right)$; and $\varepsilon_{cu}=0.003$ (Concrete crushing is considered to occur when the compressive strain in the concrete reaches its maximum allowable limit of 0.003).

The strain in the prestressing steel can be determined by applying strain compatibility, calculated as:

(14) $$\varepsilon_{ps,T}=\varepsilon_{pe,T}+\frac{P_{e,T}}{E_{c}A_{cg}}\left(1+\frac{e^{2}}{r^{2}}\right)+\varepsilon_{pnet}$$

where $\varepsilon_{pe,T}$ = effective strain in the prestressing steel after losses and $\varepsilon_{pnet}$ = net tensile strain in the prestressing steel beyond decompression, at nominal strength, depends on the failure mode and is calculated as:

(15) $$\varepsilon_{pnet}=0.003\left(\frac{d_{p}-c}{c}\right)\;\mathrm{for}\;\mathrm{concrete}\;\mathrm{crushing}\;\mathrm{failure}$$

(16) $$\varepsilon_{pnet}=\left(\varepsilon_{fe,T}+\varepsilon_{bi,T}\right)\left(\frac{d_{p}-c}{h-c}\right)\;\mathrm{for}\;\mathrm{FRP}\;\mathrm{rupture}\;\mathrm{or}\;\mathrm{debonding}\;\mathrm{failure}$$

The stress in the prestressing steel and FFP reinforcement is determined based on its temperature-dependent strength properties. For a typical seven-wire low-relaxation prestressing strand, the stress–strain relation can be approximated using the following equations.

(17) $$f_{ps,T}=\left\{\begin{array}{cc} E_{p,T}\cdot \varepsilon_{ps,T} & \mathrm{for}\;\varepsilon_{ps,T}\leq 0.0086\\ f_{u,T}-\frac{0.276}{\varepsilon_{ps,T}-0.007} & \mathrm{for}\;\varepsilon_{ps,T}>0.0086 \end{array}\right\}$$

(18) $$f_{fe,T}=E_{f,T}\cdot \varepsilon_{pe,T}$$

With the strain and stress in the FRP and prestressing steel determined for the assumed neutral axis depth, internal force equilibrium can be verified by equating compression and tensile forces.

(19) $$\alpha_{1}f_{c}^{\prime }\beta_{1}b_{T}c=A_{ps}f_{ps,T}+A_{f}f_{fe,T}$$

where α and $\beta_{1}$ are concrete stress block parameters, determined based on the parabolic stress–strain relationship for concrete, as specified in ACI 318 [26].

The neutral axis depth (c) is computed by applying force equilibrium equation and using an iterative solution process. Initially, an estimated value of (c) is assumed, and the corresponding stresses and strains are calculated using Equations (13)–(18). A revised neutral axis depth (c) is then obtained using Equation (19), and its value is compared to the initial assumption. If the computed and assumed values of (c) are consistent, the correct neutral axis depth is established. If they differ, a new (c) value is selected, and the iteration continues until convergence is achieved. Once equilibrium is established, the nominal flexural strength of the FRP-strengthened PC beam under the specified fire exposure can be determined as follows:

(20) $$M_{n,T}=A_{ps}f_{ps,T}\left(d_{p}-\frac{\beta_{1}c}{2}\right)+\psi_{f}A_{f}f_{fe,T}\left(h-\frac{\beta_{1}c}{2}\right)$$

where $\psi_{f}$ = strength reduction factor for FRP is applied to improve the reliability of strength prediction and accounts for the different failure modes observed for FRP-strengthened members (delamination of FRP reinforcement).

The failure of an FRP-strengthened PC beam at a specified fire exposure duration is evaluated by comparing its reduced moment capacity [Equation (20)] to the applied moment due to external loading. According to ASCE 7, the load level under fire conditions should be reduced below the maximum design loads used for ambient conditions, as fire is classified as an accidental event. The recommended loading for fire exposure is specified as:

(21) $$w_{fire}=1.2D+0.5L$$

where $w_{fire}$ is the loading under fire exposure and D and L are dead and live loads, respectively.

Thus, the following criterion is applied to assess the failure of an FRP-strengthened PC beam:

(22) $$M_{fire}\leq M_{n,T}$$

where $M_{fire}$ is the applied moment on the beam at the critical section during fire exposure.

The moment capacity of an FRP-strengthened PC beam and the moment due to applied loading can be determined at any specified fire exposure duration using the above set of equations. The beam is considered to have failed when the applied moment under fire conditions ($M_{fire}$) exceeds its moment capacity ($M_{n,T}$). The time at which this failure occurs defines the fire resistance of the FRP-strengthened PC beam.

## 6. Validation of the Rational Approach

The validity of the rational approach is established by comparing the predicted results with fire test data and FEA results for FRP-strengthened RC beams reported in previous studies [18,19]. Since there is a lack of fire test data in the literature for FRP-strengthened PC beams under fire exposure, validation is carried out first against an FRP-strengthened PC beam at ambient condition and then against FRP-strengthened RC beams under fire conditions. For validation under room temperature conditions, an FRP-strengthened PC beam specified in ACI 440.2R-17 [8] is analyzed using the proposed rational approach. The predicted flexural capacity at room temperature is compared with the ACI 440.2R-17 [8] as shown in Table 3. The results demonstrate a good comparison of moment capacity as well as the failure modes between the ACI and the proposed rational approach. The further validation process includes analyzing a rectangular RC beam (350 mm × 500 mm) externally strengthened with CFRP, both with and without 38 mm VG insulation, and an externally strengthened CFRP RC-T beam, both insulated and uninsulated using 19 mm Spray-Applied Fire Resistive Material (SFRM). The use of various cross-sectional geometries and fire protection strategies provide comprehensive validation of the rational approach. This validation establishes the effectiveness of the rational approach in predicting temperature distribution within the critical cross-section and assessing the resulting degradation in moment capacity caused by elevated temperatures, before extending its application to FRP-strengthened PC beams.

**Figure 6:**
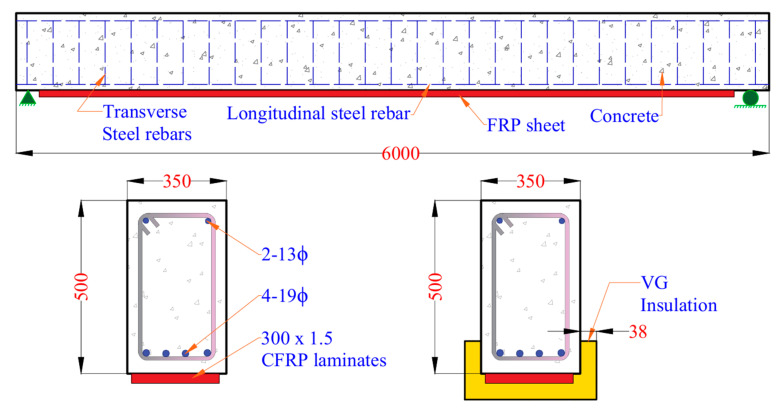
Geometry and cross-section details of CFRP-strengthened RC beams with and without VG insulation.

Figure 6 illustrates the geometry and cross-sectional details of CFRP-strengthened RC beams with and without VG insulation, based on previous research [19]. To assess the thermal behavior of these beams under fire conditions, simplified temperature prediction equations outlined in Section 3 were used to estimate temperatures at key locations: corner rebar, middle rebar, and the average temperature of the externally bonded FRP. The results obtained using the rational approach were compared with FEA data to evaluate the accuracy of the simplified method.

**Figure 7:**
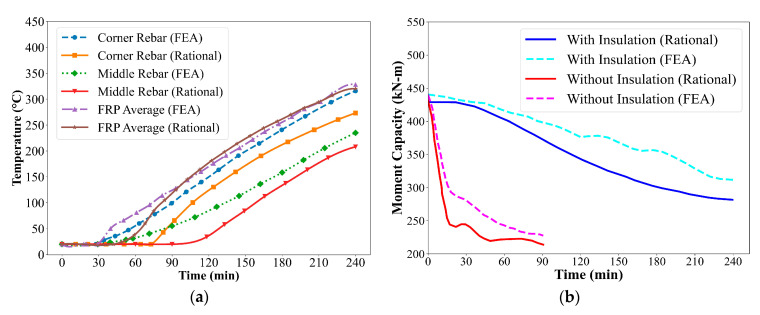
(**a**) Time–temperature profiles of CFRP-strengthened RC beam with VG insulation; (**b**) Moment capacity of CFRP-strengthened RC beam with and without insulation.

As observed in Figure 7a, FEA and rational approach exhibit consistent temperature rise trends across all monitored locations within the beam over the 240 min of ASTM E119 fire exposure. Among the three monitored positions, the average temperature in the FRP reinforcement shows the highest rise throughout the exposure duration. This is primarily due to the external placement of the FRP on the beam’s soffit, directly exposed to fire, resulting in minimal thermal insulation and a faster heat transfer rate. In contrast, the corner and middle steel reinforcement experience significantly lower temperature rise due to the presence of surrounding concrete, which acts as a thermal barrier. Overall, the rational predictions align well with the FEA results across all monitoring points, with the variation between the two methods remaining within approximately 10%. This variation is mainly attributed to the conservative assumptions adopted in the simplified temperature prediction equations and the empirical relationships used for material degradation. Nevertheless, the rational approach effectively captures the key thermal trends and provides a reliable estimation of temperature distribution in CFRP-strengthened RC beams exposed to fire.

The reduced moment capacity of CFRP-strengthened RC beams was determined by incorporating the temperature-dependent degradation of constituent material properties. Figure 7b presents the time-dependent variation in moment capacity for CFRP-strengthened RC beams with and without 38 mm VG insulation under fire exposure. Results obtained from both the rational approach and FEA show a consistent trend. For the uninsulated beam, a rapid reduction in moment capacity is observed during the early stages of fire exposure. This rapid degradation is primarily due to the direct exposure of the externally bonded FRP to elevated temperatures. As the temperature exceeds the glass transition temperature ($T_{g}$) of the polymer matrix, the resin begins to soften and decompose, significantly reducing the tensile strength contribution of the FRP to the beam’s flexural capacity. In contrast, the insulated beam exhibits a gradual decline in moment capacity over time. The presence of the 38 mm VG insulation layer significantly slows down the heat transfer to the FRP reinforcement, preserving its mechanical properties for a longer duration. The moment capacity of the insulated beam remains relatively higher throughout the 240 min fire exposure, highlighting the critical role of fire protection in enhancing structural performance.

**Figure 8:**
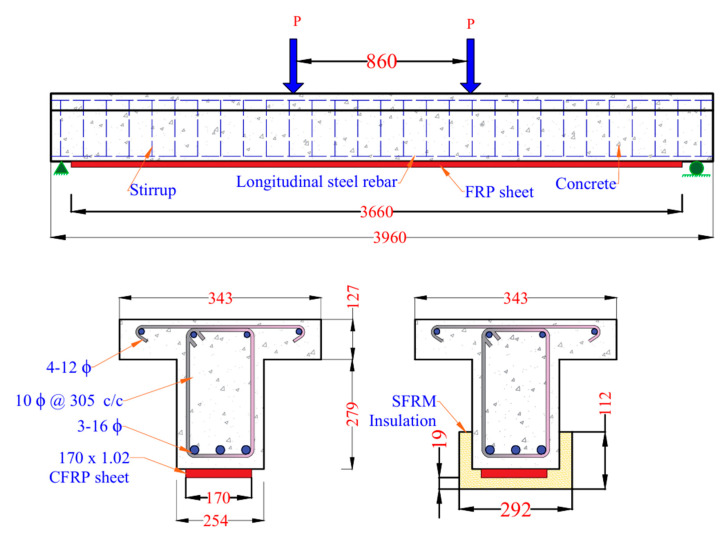
Geometry and cross-section details of CFRP-strengthened RC T-beams with and without SFRM insulation [18].

**Figure 9:**
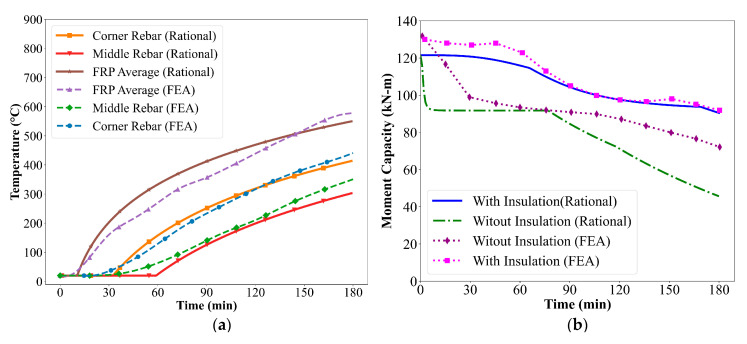
(**a**) Time–temperature profiles of CFRP-strengthened RC T-beam with SFRM insulation; (**b**) Moment capacity of CFRP-strengthened RC T-beam with and without insulation. [18].

The reliability of the rational approach was further demonstrated by comparing its predictions with the experimental and FEA results from a study on CFRP-strengthened RC T-beams tested with and without 19 mm spray-applied fire resistive material (SFRM) insulation [18]. The beam geometry and cross-sectional details are illustrated in Figure 8. The rational method closely matched the FEA results, accurately capturing the temperature rise at key locations including the FRP layer, corner reinforcement, and middle reinforcement, as shown in Figure 9a. A noticeable difference was observed between the temperature profiles of the T-beams from the study in [18] and the rectangular beams from [19]. The T-beam exhibited a faster temperature rise, primarily due to the use of a thinner insulation layer (19 mm SFRM) compared to the 38 mm VG insulation used in the rectangular beam. The rapid temperature rise in the T-beam significantly affected its structural performance under fire conditions. As the temperature exceeded the glass transition temperature of the CFRP polymer matrix, the composite began to degrade, resulting in a rapid reduction in strength. Consequently, the uninsulated T-beam lost a significant portion of its load-carrying capacity within the first 5 min of fire exposure, as illustrated in Figure 9b. In contrast, the insulated T-beam exhibited a slower and more gradual loss of capacity, as the 19 mm SFRM insulation delayed heat penetration and helped preserve the mechanical integrity of the CFRP for a longer duration.

Together, the predicted results from both studies [18,19] clearly demonstrate the effectiveness of the rational approach in estimating temperature profiles and evaluating moment capacity degradation in CFRP-strengthened RC beams under fire exposure. Although the rational predictions are close to FEA results, a slight underestimation in moment capacity is observed, mainly due to the use of simplified equations. These validation studies support the use of the rational method as a reliable tool not only for RC beams, but also for its extension to FRP-strengthened PC beams.

## 7. Parametric Studies

The rational approach has been effectively validated through comparative analysis of CFRP-strengthened RC beams, and this method can be reliably applied to evaluate the fire performance of CFRP-strengthened PC beams. In the following sub-sections, the influence of key parameters such as the presence of FRP strengthening, insulation thickness and concrete cover thickness will be discussed to assess their impact on the fire performance of FRP-strengthened PC beams.

### 7.1. Effect of Strengthening on PC Beam

**Figure 10:**
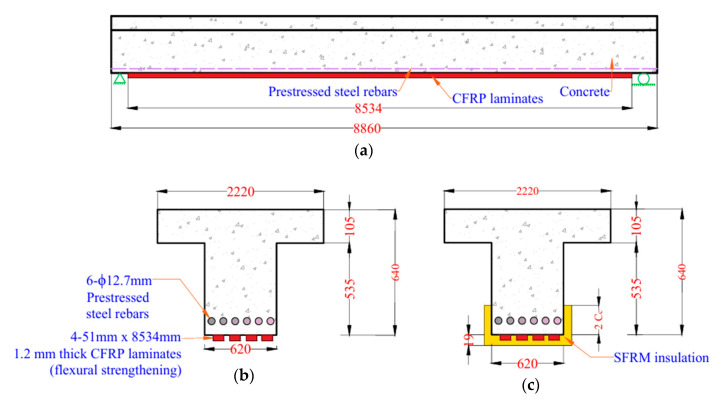
Details of CFRP-strengthened PC T-beam: (**a**) longitudinal layout, (**b**) cross-section without insulation, (**c**) cross-section with SFRM insulation.

The presence of externally bonded FRP strengthening significantly influences the fire performance of PC beams. Under ambient conditions, FRP systems enhance the flexural capacity of structural members by providing additional strength and stiffness. However, under elevated temperature exposure, the performance of FRP-strengthened beams becomes highly sensitive to thermal degradation, particularly due to the vulnerability of polymer matrix to elevated temperatures. To evaluate the effect of strengthening in fire resistance, a simply supported prestressed concrete T-beam, originally designed for a parking garage and later repurposed for office occupancy, was selected for this study. The beam is reinforced with six low-relaxation, 12.7 mm diameter, 7-wire bonded prestressing tendons. It is designed to carry a dead load of 23.8 N/mm and a live load of 19.8 N/mm, resulting in a total dead load moment of 232 kN-m. To enhance its flexural capacity, the beam is externally strengthened with CFRP laminates applied along the tension face. The flexural capacity of the strengthened PC beam is 738 kN-m. The detailed material properties and beam configurations are summarized in Table 4 and illustrated in Figure 10.

**Figure 11:**
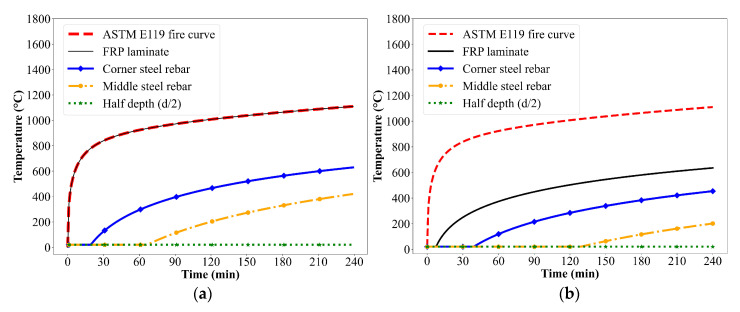
Temperature progression in CFRP-strengthened PC beam under ASTM E119 fire exposure: (**a**) without insulation and (**b**) with SFRM insulation.

The thermal and structural response of the beam under fire exposure was assessed using the rational approach at 1 min time increments throughout the duration of fire exposure, following the procedure outlined in Section 2. To determine the effective width reduction factor under fire exposure, FEA simulations were conducted using ABAQUS to generate temperature gradients across the cross-section at 15 min intervals. At each time step, concrete layers that exceeded 500 °C were excluded from the effective width calculation. The resulting reduction factors, summarized in Table 2, were subsequently used in the structural analysis. Furthermore, the temperature distribution within the cross-section of externally CFRP-strengthened PC T-beams under ASTM E119 [27] standard fire exposure, as illustrated in Figure 11, were computed using the two-dimensional heat transfer formulation (Equation (2)) developed in previous research [21].These temperature profiles were then used to evaluate the resulting degradation in material properties, including reductions in the strength and stiffness of the prestressing steel and FRP reinforcement. Based on these degraded properties, the reduced flexural capacity of the FRP-strengthened prestressed concrete beam was determined through force equilibrium and strain compatibility. Failure under fire exposure is considered to occur when this reduced flexural capacity falls below the applied moment corresponding to fire loading, with the time at which this failure occurs defining the fire resistance of the beam.

The temperature distribution within the cross-section of the CFRP-strengthened prestressed concrete T-beam subjected to ASTM E119 fire exposure is shown in Figure 11a, presenting thermal profiles at critical locations such as the FRP laminate, corner steel reinforcement, middle steel reinforcement, and the half-depth of the section (d/2). Since the FRP laminate is placed externally along the soffit of the beam, it is directly exposed to fire and exhibits a rapid temperature rise that closely follows the ASTM E119 fire curve. The FRP temperature reaches beyond 250 °C within the initial 5 min of fire exposure, at which point it loses much of its strength and stiffness. During the initial 20 min of fire exposure, the corner reinforcement remains under ambient conditions due to the insulating effect of the surrounding concrete. However, beyond this period, the corner reinforcement experiences a rapid rise in temperature, exceeding 400 °C within the first 80 min of fire exposure. At this temperature, the prestressing steel loses 50% of its tensile strength due to relaxation effects at elevated temperatures. In contrast, the middle reinforcement is subjected to comparatively lower temperatures because it is located farther from the fire-exposed surface, where heat penetration is less intense, since it is one-dimensional heat transfer. Additionally, the temperature in concrete at the half-depth remains below 20 °C throughout the fire duration, indicating minimal thermal influence in the inner concrete core.

**Figure 12:**
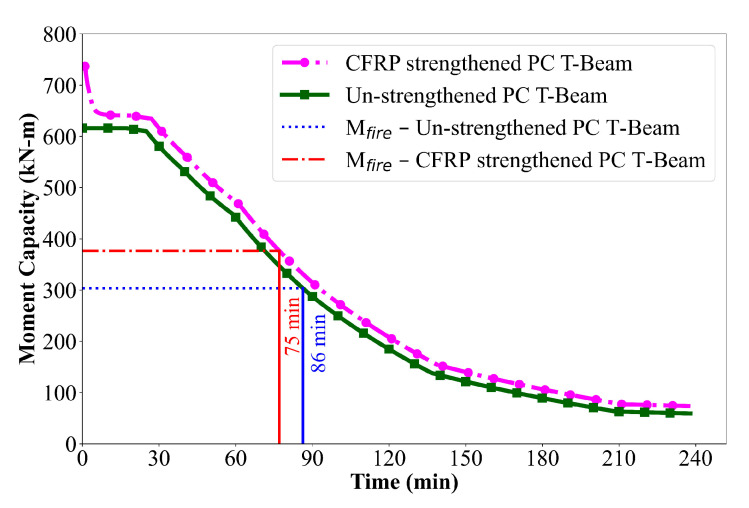
Comparison of moment capacity of PC T-beams under ASTM E119 fire exposure.

The fire resistance of un-strengthened and CFRP-strengthened prestressed concrete beams is compared in Figure 12, which shows the variation in moment capacity over time under ASTM E119 standard fire exposure. In the case of the un-strengthened PC beam, the moment capacity gradually declines with increasing fire exposure duration. During the first 30 min, the beam maintains most of its original capacity, primarily due to inherent thermal resistance of concrete, which delays the penetration of heat to the core of the section and provides some thermal protection to the prestressing tendons. However, after 30 min, a noticeable loss in capacity is observed, which can be attributed to the rapid degradation in the strength of the prestressing steel resulting from a temperature rise beyond 250 °C. In addition, the compressive strength of concrete in the outer zones begins to deteriorate as thermal exposure progresses, contributing to the reduction in moment capacity. At approximately 86 min, the combined degradation of strength in the prestressing steel and concrete causes the reduced flexural capacity to fall below the applied moment under fire loading, resulting in failure.

In contrast, the CFRP-strengthened PC T-beam exhibits a distinctly different response under fire conditions compared to the un-strengthened beam (see Figure 12). The initial moment capacity is significantly higher due to the additional strength and stiffness contribution provided by the externally bonded CFRP laminates. However, the strengthened beam undergoes a much faster rate of strength degradation during fire exposure. A sharp decline in moment capacity is observed within the initial 5 min, primarily because the FRP laminate is directly exposed to fire and is highly sensitive to elevated temperature exposure. Once the temperature at the FRP level begins to exceed the glass transition temperature of the polymer matrix, the laminate begins to soften and rapidly lose its tensile capacity. As a result, the moment capacity of the beam drops quickly and approaches that of the un-strengthened beam.

Despite this rapid loss, the strengthened beam retains slightly higher capacity throughout the fire exposure period due to cable action provided by the CFRP. Failure occurs at approximately 75 min, which is less than the un-strengthened beam. This is mainly due to the higher initial moment capacity resulting from CFRP strengthening which causes the reduced flexural capacity to fall below the applied moment corresponding to fire loading ($M_{fire}$). As the CFRP rapidly loses its tensile capacity at elevated temperatures, the beam is unable to sustain the increased demand, leading to earlier failure.

### 7.2. Effect of Fire Insulation

Fire insulation is essential for enhancing the fire resistance of structural members, especially those incorporating temperature-sensitive materials such as FRP. As demonstrated in Figure 12, the absence of insulation leads to rapid thermal degradation of the CFRP laminate, resulting in a substantial reduction in structural capacity and earlier failure. This highlights the critical role of insulation in maintaining the effectiveness of CFRP-strengthened beams under elevated temperatures, where unprotected systems become susceptible to premature failure despite their superior strength in ambient conditions. SFRM insulation was used in this study to protect the externally bonded CFRP laminate from direct fire exposure. This type of insulation was shown to be effective for RC members strengthened with FRP, as demonstrated in previous research [28].As shown in Figure 10c, the insulation was applied in a U-shaped configuration along the soffit and sides of the beam to delay heat penetration and enhance fire resistance. For the initial analysis, a 19 mm thick SFRM insulation layer was considered, with its material properties summarized in Table 4.

The temperature distributions within the cross-section of externally CFRP-strengthened PC T-beams with 19 mm SFRM insulation, exposed to ASTM E119 standard fire conditions, were computed using a two-dimensional heat transfer analysis, as illustrated in Figure 11b. This was achieved by converting the insulated section into an equivalent concrete section, based on the formulation developed in previous research [21] and defined by Equations (6) and (7). Due to the presence of SFRM insulation, a significant reduction in temperature is observed at all monitored locations compared to uninsulated beams. The temperature at the FRP laminate remains at ambient condition for an initial 10 min of fire exposure. This initial thermal resistance is due to the low thermal conductivity and high specific heat capacity of the insulation material, which effectively slows down heat transfer. As fire exposure continues, the temperature at the FRP gradually increases but remains below 700 °C even after 240 min, demonstrating the sustained insulating effect that significantly reduces the rate of thermal penetration. Similarly, the corner prestressing steel rebar remains at room temperature level for about 40 min. This delayed heating is due to the protective effect of both the SFRM insulation and the effective concrete cover, which collectively act as a thermal barrier. As the fire exposure continues, the temperature at this location rises but remains below 500 °C at the end of the 240 min fire duration. In contrast, the middle rebar and concrete at half the section depth (d/2) experience minimal temperature increase and remain close to ambient temperature level for the majority of the fire exposure period. This is due to the farther location from the fire-exposed surface, resulting in very limited thermal impact throughout the fire exposure.

**Figure 13:**
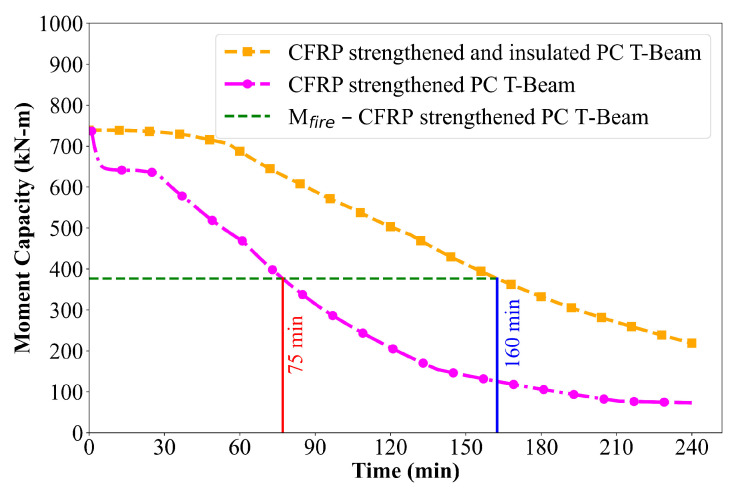
Moment capacity degradation of CFRP-strengthened and insulated PC T-beams under ASTM E119 fire exposure.

The reduced temperature levels observed in the insulated CFRP-strengthened beam directly influence its structural performance under fire exposure. As illustrated in Figure 13, the moment capacity of the strengthened and insulated beam remains significantly higher and degrades more gradually compared to uninsulated strengthened beam. The presence of insulation effectively preserves the strength and stiffness contributions of both the CFRP and the prestressing steel over an extended duration, resulting in a slower decline in flexural capacity. Notably, the insulated beam sustains its load-carrying capacity for up to 160 min, as the insulation slows heat transfer and helps to preserve the mechanical properties of both the CFRP and the prestressing steel, which is more than twice the fire resistance of the uninsulated CFRP-strengthened beam. These results highlight the critical role of fire insulation in enhancing both the thermal protection and structural reliability of FRP-strengthened PC members during fire exposure.

To further evaluate the effectiveness of fire insulation, a parametric study was conducted by varying the insulation thickness, while keeping the beam geometry, material properties, and other parameters unchanged. In addition to the baseline beam with 19 mm insulation, four other SFRM insulation thicknesses were considered: 0 mm, 12.5 mm, 16.7 mm, and 25 mm. As shown in the results presented in Table 5, increasing the insulation thickness significantly improves the fire resistance of the CFRP-strengthened PC beam. Notably, with 25 mm of insulation, the beam reached a failure time of 201 min, indicating that this thickness is sufficient to achieve approximately 3 h of fire resistance under ASTM E119 standard fire exposure.

### 7.3. Effect of Concrete Cover Depth

Concrete cover depth is a critical parameter that significantly influences the fire performance of CFRP-strengthened PC beams. It primarily influences the rate of heat transfer to the internal reinforcement, thereby affecting both the thermal response and structural performance under elevated temperatures. In this study, the effect of concrete cover depth was examined by considering two configurations: 50 mm and 64 mm. The 50 mm cover was selected in accordance with the provisions of ACI 216.1-14 [29], which specify that a minimum cover thickness of 50 mm is required for prestressed concrete beams to achieve a 2 h fire resistance rating. All other parameters, including beam geometry, material properties, insulation thickness, and loading conditions, were kept unchanged to assess the influence of cover depth. The results presented in Table 5 indicate that increasing the concrete cover depth enhances the fire resistance of both un-strengthened and CFRP-strengthened beams. A greater effective cover depth provides improved thermal insulation to the embedded prestressing tendons, delaying their exposure to elevated temperatures and reducing the rate of material degradation. Specifically, for the insulated CFRP-strengthened beam, increasing the cover depth from 50 mm to 64 mm resulted in an increase in failure time from 133 min to 160 min. This improvement is attributed to the additional concrete mass, which acts as a thermal barrier and reduces the rate of heat conduction into the concrete core during fire exposure.

## 8. Limitations

The applicability of the proposed rational approach is subject to certain limitations, as it is validated only for FRP-strengthened PC beams under specific conditions.

- The rational approach assumes no relative slip between the external FRP reinforcement and the concrete; however, this assumption may not be fully valid in practical applications, where relative slip can occur under high temperature exposure. Thus, this limitation may not be critical in the core region of the beams.
- Although the rational approach has been validated for various FRP-strengthened RC beams, further validation against fire test data of FRP-strengthened PC beams can enhance the validity of the model.
- The rational method for temperature evaluation is restricted to standard fire scenarios, including ASTM E119 and ISO 834. These equations may have to be slightly revised to the real design fire conditions.
- The temperature predictions for FRP-insulated PC beams do not account for uncertainties such as cracking, uneven insulation thickness, or moisture evaporation in concrete and insulation, which can have some minor influence on the actual cross-sectional temperature distribution.
- The rational approach does not consider the effect of fire-induced axial restraints in PC beams. In practical conditions, axial restraints can develop due to thermal expansion and boundary conditions, affecting structural response and fire resistance.
- Concrete spalling is not considered in the proposed approach. However, its impact is minimal, as spalling is less likely to occur in normal-strength concrete subjected to standard fire conditions.
- The proposed rational approach is limited to normal-strength concrete and does not account for the fire behavior of high-strength concrete, which is more susceptible to spalling and may exhibit different thermal and mechanical degradation characteristics.
- Shear failure is not considered in the proposed approach, as flexural behavior predominantly governs in FRP-strengthened members; however, it can be incorporated with slight modifications to the proposed approach.
- The long-term performance of FRP-strengthened RC and PC beams under sustained service conditions and elevated temperatures is not considered in this study. As fire exposure may influence creep, relaxation, and durability effects of FRP systems, future research is needed to evaluate long-term behavior.

## 9. Conclusions

Based on the findings of this study, the following conclusions can be drawn:

- The proposed rational approach demonstrates effectiveness in evaluating the fire resistance of CFRP-strengthened PC beams, with validation against CFRP-strengthened RC beams. The rational methodology predictions closely match FEA results, with deviations remaining within an acceptable range of ±10%.
- The CFRP-strengthened PC T-beam, despite its higher initial capacity, fails earlier (75 min) than the un-strengthened PC T-beam (86 min) due to rapid degradation of externally bonded CFRP at elevated temperatures, which highlights the significant fire risk of unprotected FRP systems.
- The fire resistance of FRP-strengthened PC beams can be significantly enhanced through supplementary fire insulation. The parametric study reveals that at least 25 mm of insulation is required to achieve 3 h fire resistance for FRP-strengthened PC T-beams.
- The thickness of the concrete protective cover also affects fire performance, though its influence is secondary to that of insulation. An increase in cover depth from 50 mm to 64 mm extended the failure time by approximately 20–30 min, primarily by delaying heat transfer to the prestressing tendons.

This study provides a foundation for evaluating the fire resistance of FRP-strengthened PC beams using a rational approach that incorporates temperature-dependent material degradation and insulation effects. While validated against available RC beam data and applied to selected PC beam configurations, the findings highlight critical insights into the influence of strengthening, insulation thickness, and cover depth on FRP-strengthened PC beams in fire exposure. Future work can build on this by conducting fire tests on FRP-strengthened PC beams and incorporating additional effects such as shear behavior, axial restraint, fire induced spalling and real fire scenarios to further validate and expand the applicability of the proposed approach.

## Figures and Tables

**Table 1:** Factors for temperature evaluation in concrete members [21].

Factor	ASTM E119		ISO 834	
a	910		935	
n	0.148		0.168	
	NSC-carbonate	NSC-silicate	HSC-carbonate	HSC-silicate
$c_{1}$	1.0	1.01	1.12	1.12
$c_{2}$	1.0	1.06	1.12	1.20

NSC—normal-strength concrete. HSC—high-strength concrete.

**Table 2:** Effective width reduction factors of PC T-Beam under ASTM E119 fire exposure.

Time (min)	Effective Width Factor	Time (min)	Effective Width Factor
0	1.000	135	0.931
15	1.000	150	0.927
30	0.999	165	0.909
45	0.981	180	0.898
60	0.977	195	0.891
75	0.962	210	0.882
90	0.956	225	0.859
105	0.948	240	0.837
120	0.939		

**Table 3:** Comparison of moment capacity based on ACI 440.2R-17 [8] and rational approach.

Beam Designation	$f_{c}^{\prime }$ (MPa)	$A_{ps}\;\left({\mathrm{mm}}^{2}\right)$	$A_{f}\;\left({\mathrm{mm}}^{2}\right)$	Failure Mode	$\mathrm{Moment}\;\mathrm{Capacity}\;(M_{n}$) (kN-m)	
					ACI 440.2R-17	Rational Approach
PC	27.6	495	-	Prestressing steel yield	505	505
PC-CFRP	27.6	495	610	FRP debonding	636	636

**Table 4:** Material Properties of CFRP-Strengthened PC T-beam.

Parameter	Value
Concrete compressive strength ($f_{c}^{\prime })$	41.4 MPa
Effective prestress in tendon ($f_{pe})$	1138 MPa
Yield strength of prestressing steel $\left(f_{py}\right)$	1586 MPa
Ultimate strength of prestressing steel $\left(f_{pu}\right)$	1860 MPa
Modulus of elasticity of prestressing steel $\left(E_{p}\right)$	1.96 × 10^5^ MPa
Ultimate tensile strength of FRP $\left(f_{fu}\right)$	2800 MPa
Rupture strain of FRP $\left(\varepsilon_{fu}\right)$	0.017
Modulus of elasticity of FRP laminates $\left(E_{f}\right)$	1,600,000 MPa
Insulation thermal conductivity $\left(k_{i}\right)$	0.154 W/m-K
Insulation specific heat ($c_{i}$)	1.888 kJ/kg-K
Insulation density $\left(\rho_{i}\right)$	425 kg/m^3^

**Table 5:** Parametric study on PC T-beam under ASTM E119 fire exposure.

Beam Designation	Effective Cover (mm)	Insulation (mm)	Failure Time (min)			Variable
			Un-Strengthened Beam	Strengthened Beam	Strengthened and Insulated Beam	
T1	50	19.0	61	54	133	Effective cover
T2	64	19.0	86	75	160	Effective cover
T3	64	0.0	86	75	75	No Insulation
T4	64	12.5	86	75	124	Insulation
T5	64	16.7	86	75	147	Insulation
T6	64	25.0	86	75	201	Insulation

## Data Availability

Data presented in the paper will be made available upon reasonable request.
